# The Impact of Peripheral Vascular Motion on Acute Drug Retention of Intravascular Devices

**DOI:** 10.1007/s13239-025-00776-z

**Published:** 2025-02-13

**Authors:** Trey Ursillo, Kayla Lowry, Catherine Allred, Mollie Phillips, Linda B. Liu, Danyi Chen, Saami K. Yazdani

**Affiliations:** Department of Engineering, Wake Forest University, Winston-Salem, NC 27101 USA

**Keywords:** Drug delivery, Peripheral artery disease, Bioreactor, Paclitaxel, Drug retention, Vascular motion

## Abstract

**Purpose:**

This goal of this study was to determine the impact of vascular motion on acute drug transfer and retention of drug-coated balloons (DCB) or drug-eluting stents (DES).

**Methods:**

Commercially available paclitaxel DCBs (Lutonix & IN.PACT) and a paclitaxel DES (Zilver) were subjected to physiological flow and vascular motion conditions using a peripheral-simulating benchtop bioreactor system. Each DCB- or DES-treated artery was subjected to three sets of movement parameters including pulsatile flow with no twisting/bending (P1), pulsatile flow with 16.8° twist, 25° bend and 3.2 mm compression (P2), and pulsatile flow with 68° twist, 35° bend, 21 mm compression (P3). After 24 h, the treated segments were removed and paclitaxel concentrations were measured using pharmacokinetic analysis.

**Results:**

In the group of arteries treated with the Lutonix DCB, there was a significant decrease in arterial paclitaxel concentrations between the P1 and both the P2 and P3 moving parameters (P1 = 404 ± 195 ng/mg, P2 = 14.9 ± 9.92 ng/mg, P3 = 19.2 ± 15.4 ng/mg; P1-P2 *p* = 0.007, P1-P3 *p* = 0.005). For the IN.PACT DCB group, no differences in the mean arterial paclitaxel concentrations were observed for the various movements (*p* = 0.55). Lastly, in the Zilver DES group, differences were only measured between the P2 and P3 moving parameters (P2 = 84.8 ± 32.7 ng/mg, P3 = 0.11 ± 0.06 ng/mg; P2-P3 *p* = 0.01).

**Conclusion:**

Acute retention of arterial paclitaxel levels can be adversely impacted by vascular movement in both DES- and DCB- treated arteries.

## Introduction

Peripheral artery disease (PAD) is the narrowing of arteries in the extremities, normally due to the buildup of fatty plaque, and is a major health concern worldwide with a prevalence of over 200 million [1]. Historically, methods for treating PAD included endovascular approaches such as stenting with bare metal stents (BMS) and percutaneous transluminal angioplasty (PTA) [2]. Both stenting and PTA break apart and push fatty plaque into the arterial wall, thereby widening the artery and reducing risk of stroke and heart attack [3]. Although endovascular approaches are becoming increasingly preferred over more invasive surgeries, up to 31% of patients who undergo stenting or balloon angioplasty treatments for PAD experience restenosis of the artery within 3 months [4]. Restenosis occurs largely in part due to damage sustained to the arterial wall during treatment of PAD, which initiates the proliferation of smooth muscle cells, a process known as neointimal hyperplasia [5]. Prevention of neointimal hyperplasia is largely achieved through drug-coated devices, namely, drug-coated balloons (DCB) and drug-eluting stents (DES) [6]. Most DCB and DES utilize local delivery of paclitaxel, a lipophilic antiproliferative drug that is easily absorbed into the arterial wall and inhibits smooth muscle cell proliferation through altering microtubule assembly [7]. As a result, DCB and DES have become the gold standard compared to BMS and PTA for treating atherosclerosis, especially in regard to minimizing the risk of restenosis due to intimal hyperplasia [8, 9].

One area that is frequently affected by PAD is the femoropopliteal artery (FPA), which is the most common site of lower extremity atherosclerosis [10]. The FPA starts at the superficial femoral artery (SFA) and runs from the upper thigh down to the popliteal artery (PA) which continues behind the knee [11]. Due to its anatomical placement, the FPA frequently moves due to various normal activities such as sitting, walking, or climbing stairs. These movements have significant effects on the FPA, including shortening in the axial direction, twisting, and bending [12]. These effects are even more pronounced in the PA due to its placement behind the knee, which results in more significant changes in length, curvature, and twist compared to the SFA [13, 14]. As a result, rates of stent fracture are significantly higher in the FPA compared to other vessels affected by PAD [15], which has the potential to reduce patency [16]. In contrast, treatment of PAD in the FPA using DCB is generally effective in reducing late lumen loss and restenosis in the short term [17, 18]. Despite evidence that the treatment of PAD in the FPA using DES results in worsened long-term patency due to vascular motion in comparison to treatment with DCB, no study has examined how such movement might affect the absorption and retention of drugs in the affected area. In this study, we investigated the effects of vascular motion on acute drug retention for both DCB and DES using an ex-vivo peripheral simulating bioreactor system.

## Methods and Materials

### Vessel Harvest and Bioreactor Systems

Harvested porcine carotid arteries from local abattoirs were rinsed in sterile PBS, and the excess fat, connective tissue, and fascia were dissected and removed. Vessels were then stored in 15mL centrifuge tubes and frozen at − 20 °C until needed. Before the arteries were placed into the bioreactor, they were thawed in a 15 mL mixture containing 10 mL of DMEM (low glucose [1000 mg/L], 4.0 mmol/L L-glutamine, 110 mg/L sodium pyruvate, pyridoxine hydrochloride, 10% fetal bovine serum (Gibco), and 1% antibiotic-antimycotic (Gibco)) and 5 mL of antibiotic-antimycotic (Gibco).

**Fig. 1:**
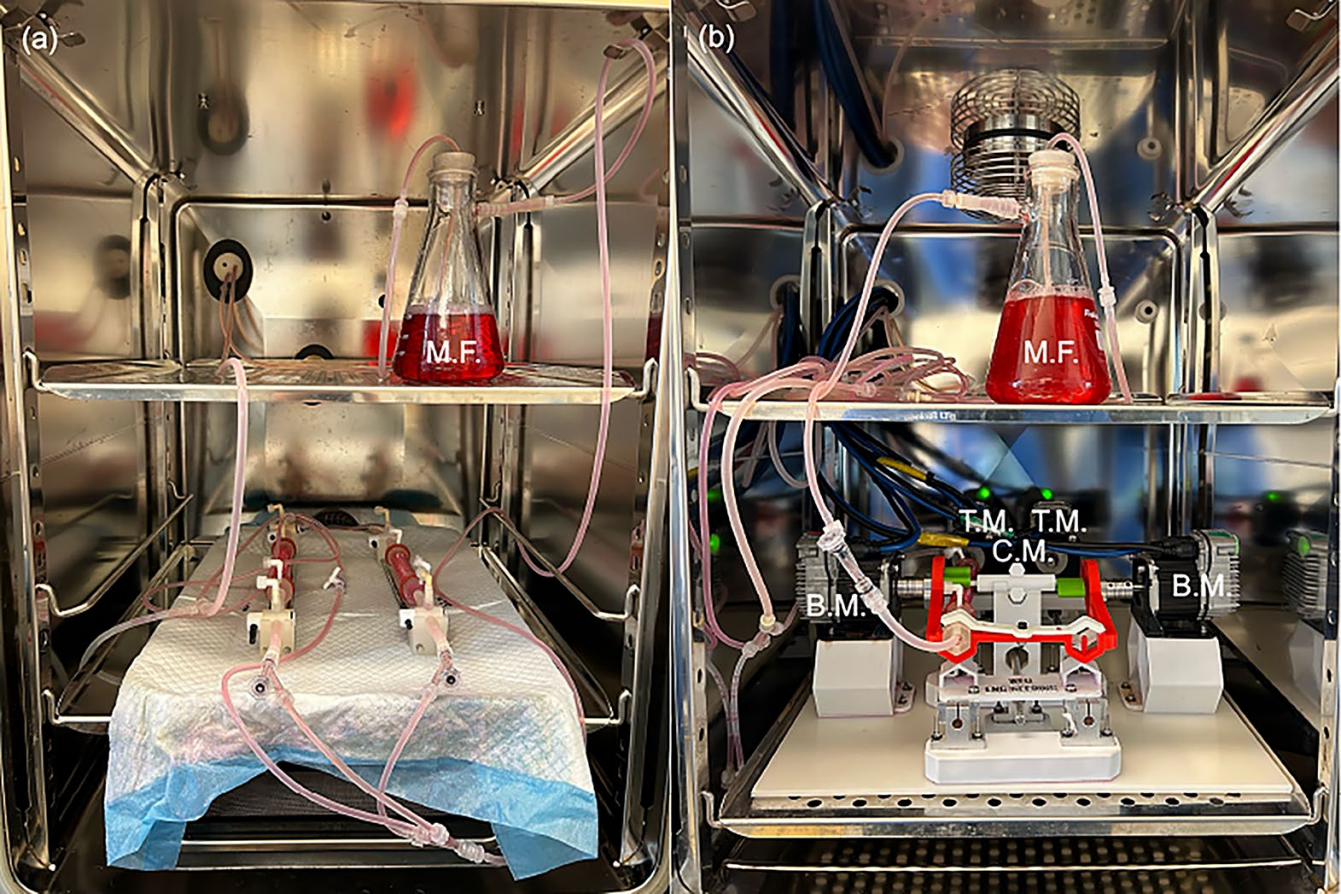
Ex vivo bioreactor setups. Operational view of the (**a**) pulsatile flow only and (**b**) the peripheral simulating bioreactor located within CO_2_ incubators. Both systems incorporate a medial flask (M.F.) and the peripheral simulating bioreactor incorporates a bending motor (B.M.), twisting motor (T.M.) and a compression motor (C.M.) to lengthen and shorten the harvested porcine carotid arteries

To evaluate the impact of vascular motion on drug retention, two bench-top models were utilized. The first was a stationary bioreactor setup that only exposed the harvested carotid arteries to pulsatile flow and pressure (Fig. 1a). The flow system consisted of a gear pump (Ismatec, Cole-Parmer, Vernon Hills, IL) a flow reservoir, a distal flow restrictor, and two flexible vessel housing compartments made from silicone. The bioreactor used approximately 350 mL of flow medium consisting of DMEM with the same composition as described above. The bilateral design of this flow bench-top model allowed for the evaluation of two vessels simultaneously. The flow rate and pressure were monitored via an ultrasonic flow meter (Transonic Systems Inc., Ithaca, NY) and a catheter pressure transducer (Millar Instruments, Houston, TX).

The second setup, the peripheral simulating bioreactor system, consisted of two primary components: a flow system and an actuating system (Fig. 1b). The flow system is identical to the stationary bioreactor setup previously described. The actuating system is constructed using custom 3D-printed components and off-the-shelf parts. The movement of the vessels within the bioreactor is controlled by five Teknic Clearpath-SCSK integrated DC servo motors, each with a peak torque output of 0.5 Nm and a power rating of 75 W. Two motors are mounted axially to each vessel to apply torsional force, while two additional motors are mounted perpendicular to the vessels to facilitate bending around a removable mandrel. A single motor actuates a linear drive, which simultaneously applies axial shortening (compression) to all vessels. The bioreactor allows for a range of motion in three axes: twisting, bending, and shortening-elongation. The twisting motion has a range of 0° to 180°, while the bending motion can be achieved within a range of 0° to 90° with a radius of 5 to 300 mm. The shortening-elongation motion has a range of 0 to 100 mm. The peripheral simulating bioreactor operates cyclically, with motors applying flexion, torsion, and compression forces over a 6 s interval before reversing to the starting position. The system then maintains this position for 4 s until the next cycle is activated, for a total of 10 s per cycle. These cycles are designed to simulate natural human movements throughout the day, such as walking, sitting, or climbing stairs. A more comprehensive description of the peripheral simulating bioreactor’s system architecture has been described previously in the literature [19].

### Experimental Setup and Deployment of DCBs and DES

**Fig. 2:**
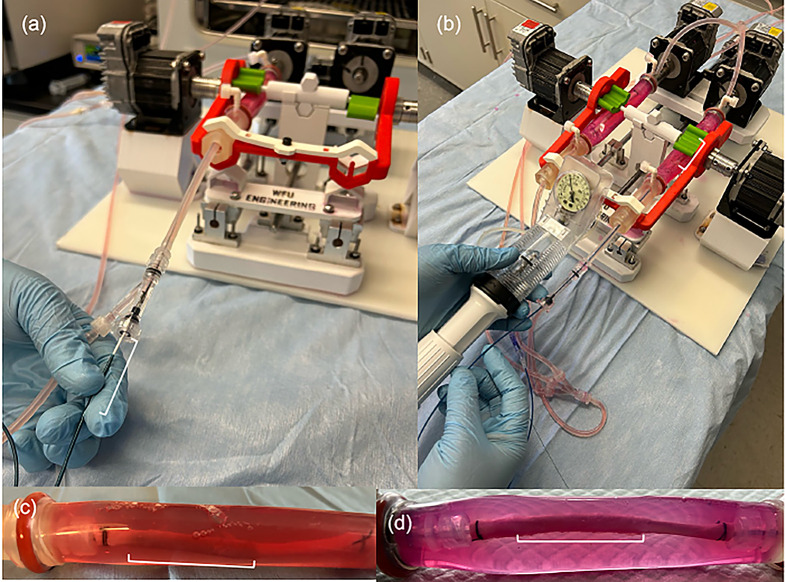
Deployment of intravascular devices into the peripheral simulating bioreactor system. (**a**) Insertion of the DES and DCB into the circulatory system occur through a Y-luer fitting adapter. (**b**) Following positioning of the devices, the DES and DCB are deployed using an indeflator at the manufacturer’s recommendation pressure. Post deployment view of (**c**) the DES and (**d**) deployment of the DCB within the harvested porcine carotid arteries

**Fig. 3:**
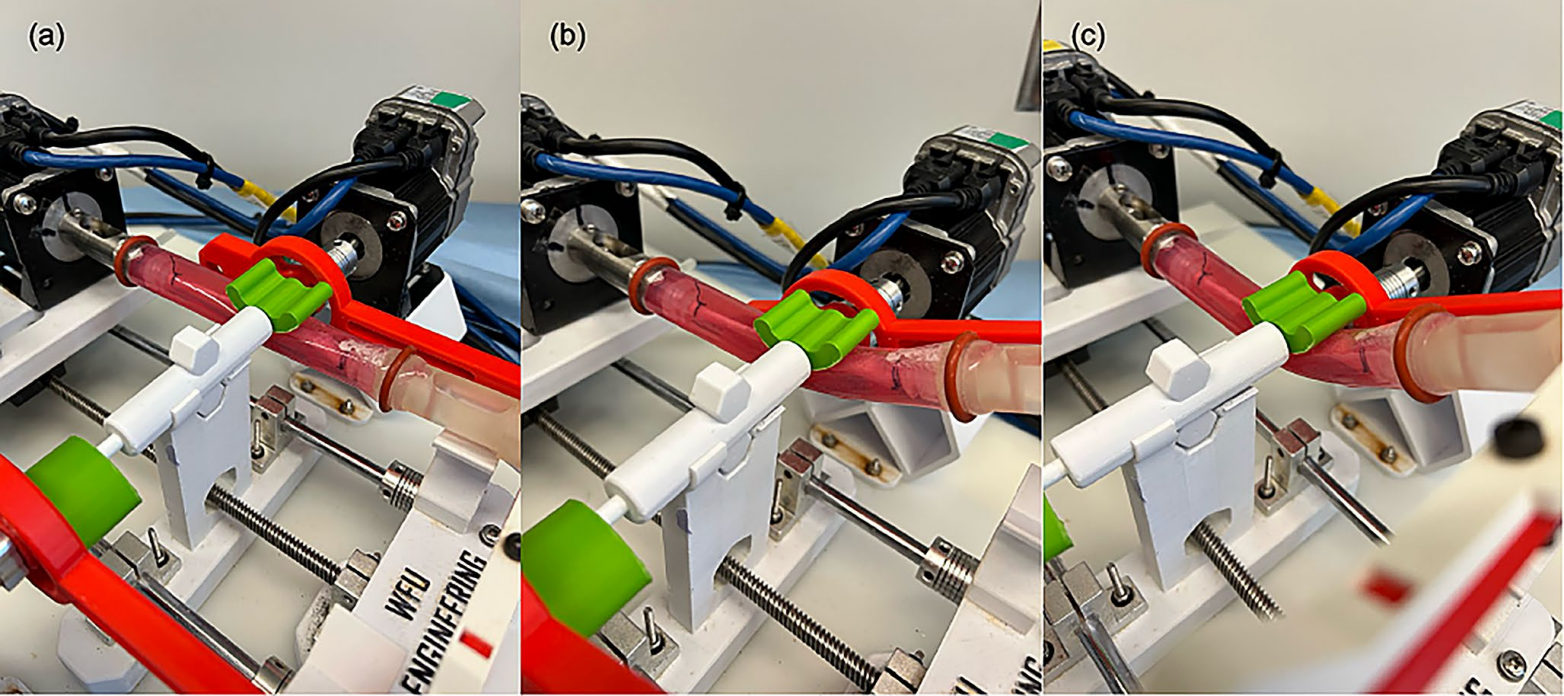
Movement of the artery under the various movement parameters. (**a**) Parameter 1, P1: Pulsatile flow with no twisting, bending, or compression. (**b**) Parameter 2 (P2): Pulsatile flow with 16.8° twist, 25° bend, 3.2 mm compression. (**c**) Parameter 3 (P3): Pulsatile flow with 68° twist, 35° bend, 21 mm compression

To simulate the effect of movement on acute drug retention in the popliteal artery, two commercially available DCBs (Lutonix, BD; IN.PACT, Medtronic) and one DES (Zilver, Cook Medical) were deployed in the harvested porcine carotid arteries using the benchtop bioreactor flow systems in a CO_2_ incubator. Prior to delivery, a guidewire was positioned within the lumen of the artery. Following endothelial injury by balloon angioplasty, the diameter of the explanted artery was measured by ultrasound. The DCB or DES of choice was then deployed into the artery based on the manufacturer’s delivery parameter recommendation (Fig. 2). For the DCBs, the inflation was for a duration of 2 min. After deployment, the DES was left inside the artery, and the DCB was removed. Fig. 2 shows representative images of the DCBs and DES being deployed within the porcine carotid arteries of the stationary and peripheral-simulating ex vivo bioreactor systems (Fig. 3).

After 2 h of pulsatile-only flow conditions, the arteries were subjected to one of three sets of movement parameters: (1) Parameter 1 (P1): Pulsatile flow with no twisting, bending, or compression; (2) Parameter 2 (P2): Pulsatile flow with 16.8° twist, 25° bend, 3.2 mm compression; and (3) Parameter 3 (P3): Pulsatile flow with 68° twist, 35° bend, 21 mm compression. The values for each movement parameter were selected based on the measured conformational change of the FPA when exposed to different body positions [12, 13].

In all models, the treated arteries were exposed to a simulated physiological flow consisting of a mean flow rate of 70 mL/min and a systolic/diastolic pressure of 90/25 mmHg. The culturing media was replaced after 2 h and fresh antibiotics added at 2 and 12 h. At 24 h post-deployment, the pulsatile flow was stopped and the arteries were harvested for pharmacokinetic (PK) analysis. Due to the optical clarity of the vessel housing, the treatment area could be clearly identified to ensure that only the portion of the artery treated by the drug was excised (Fig. 2c and d). The treated portion of the artery was cut into 3 equal sections and PK analysis was performed on the center section.

### Post-Deployment Quantification of Paclitaxel Tissue Concentrations and Analysis of Arterial Stent Positioning and Structure

Tissue concentrations of paclitaxel were measured using a high-performance liquid chromatography (HPLC) system coupled with electrospray ionization and tandem mass spectrometry (LC–MS/MS). This validated assay method has been previously described in the literature [20].

To visualize the stent placement inside the artery after exposure to movement, one artery treated with a Zilver stent from both the P2 and P3 movement groups were fixed with 10% formalin and subsequently imaged by micro-computed tomography (Micro CT). The specimens were imaged with X-ray Micro CT Nikon XTH 225 ST PE1621 EHS system using a Tungsten target at 105 keV and 100 µA with a 0.500 mm copper filter using 1200 projections of 500 ms each resulting in a 20 min scan with a resolution of 35.9 μm. To examine the stent surface in greater detail after Micro CT, the stents were removed from the artery and were mounted and sputter-coated with gold. The specimens were visualized using a Phenom XL scanning electron microscope (SEM; Thermo Fisher Scientific, Waltham, MA).

### Statistical Analysis

All data are expressed as mean ± standard deviation (SD). Continuous variables were compared between groups using one-way analysis of variance (ANOVA) using GraphPad Prism 9 (GraphPad Software, La Jolla, CA, USA). A value of *p* ≤ 0.05 was considered statistically significant. Tukey’s multiple comparisons post hoc test was used to identify differences between groups if statistical differences were found with ANOVA.

## Results

### Pharmacokinetic Analysis

**Fig. 4:**
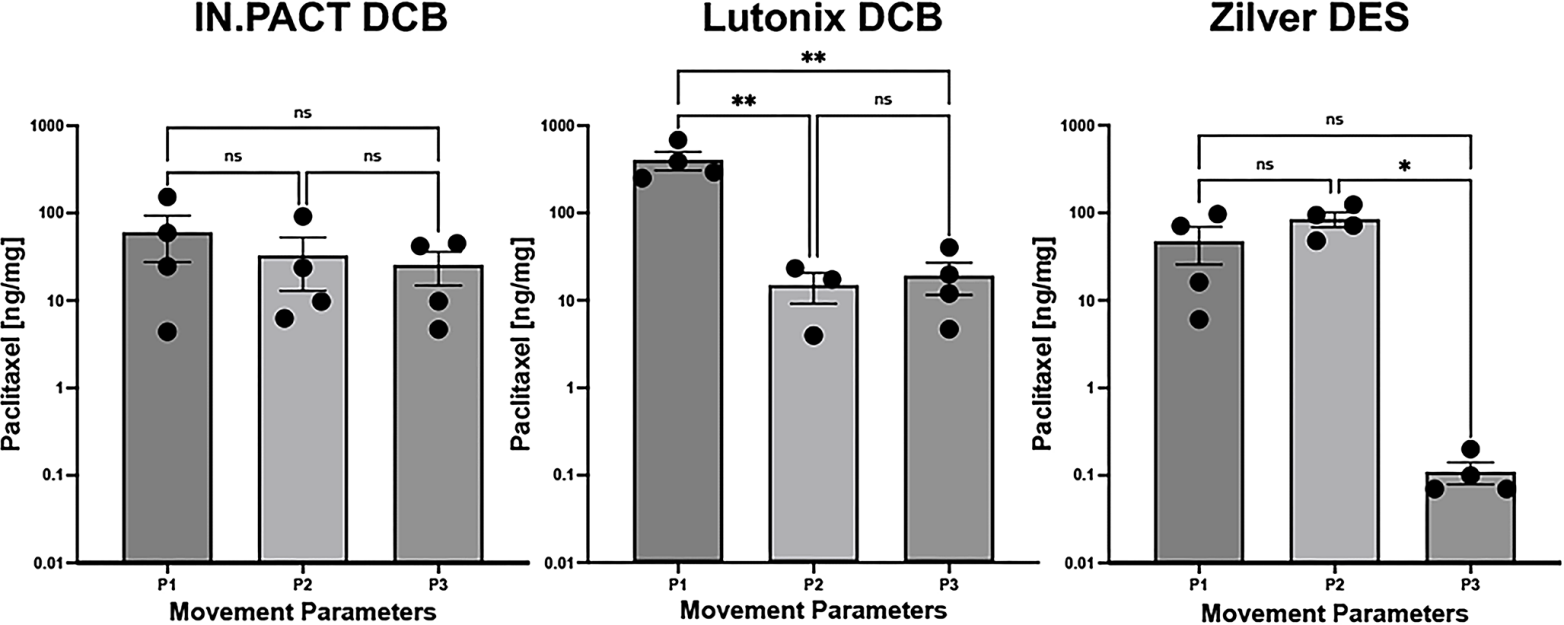
Mean concentration of paclitaxel for each DCB/DES under different movement parameters at 24-hours. Data is presented using a log scale for visualization purposes. Each dot represents a biological repeat. Error bars represent standard error mean. P1 (Parameter 1): Pulsatile flow with no twisting, bending, or compression; P2 (Parameter 2): Pulsatile flow with 16.8° twist, 25° bend, 3.2 mm compression; P3 (Parameter 3): Pulsatile flow with 68° twist, 35° bend, 21 mm compression. Pharmacokinetic data were analyzed using one-way ANOVA with Tukey’s multiple comparisons post hoc test. (ns – not significant, ∗ – p < 0.05, ∗∗ – p < 0.01)

To evaluate tissue drug retention, treated arterial segments were removed 24 h post-deployment. In the peripheral-simulating 24 h groups, a total of 7,920 cycles at the prescribed twist, elongation and axial shortening were completed, which is in proximity to the average number of steps taken per day by the average U.S. adult [21, 22]. In arteries treated with the IN.PACT DCB, no differences in the mean arterial paclitaxel concentrations were observed for the various movements (P1 = 60.5 ± 66.0 ng/mg, P2 = 32.8 ± 39.8 ng/mg, P3 = 25.5 ± 21.2 ng/mg; *p* = 0.55, Fig. 4A). Within the Lutonix DCB group, there was a significant decrease in arterial paclitaxel concentrations between the P1 and both the P2 and P3 moving parameters (P1 = 404 ± 195 ng/mg, P2 = 14.9 ± 9.92 ng/mg, P3 = 19.2 ± 15.4 ng/mg; P1-P2 *p* = 0.007, P1-P3 *p* = 0.005, Fig. 4B). Lastly in the Zilver DES group, differences were only detected between the P2 and P3 moving parameters (P1 = 47.5 ± 43.6 ng/mg, P2 = 84.8 ± 32.7 ng/mg, P3 = 0.11 ± 0.06 ng/mg; P1-P3 *p* = 0.14, P2-P3 *p* = 0.01, Fig. 4 C).

### Micro CT and SEM of Stents in the Artery

**Fig. 5:**
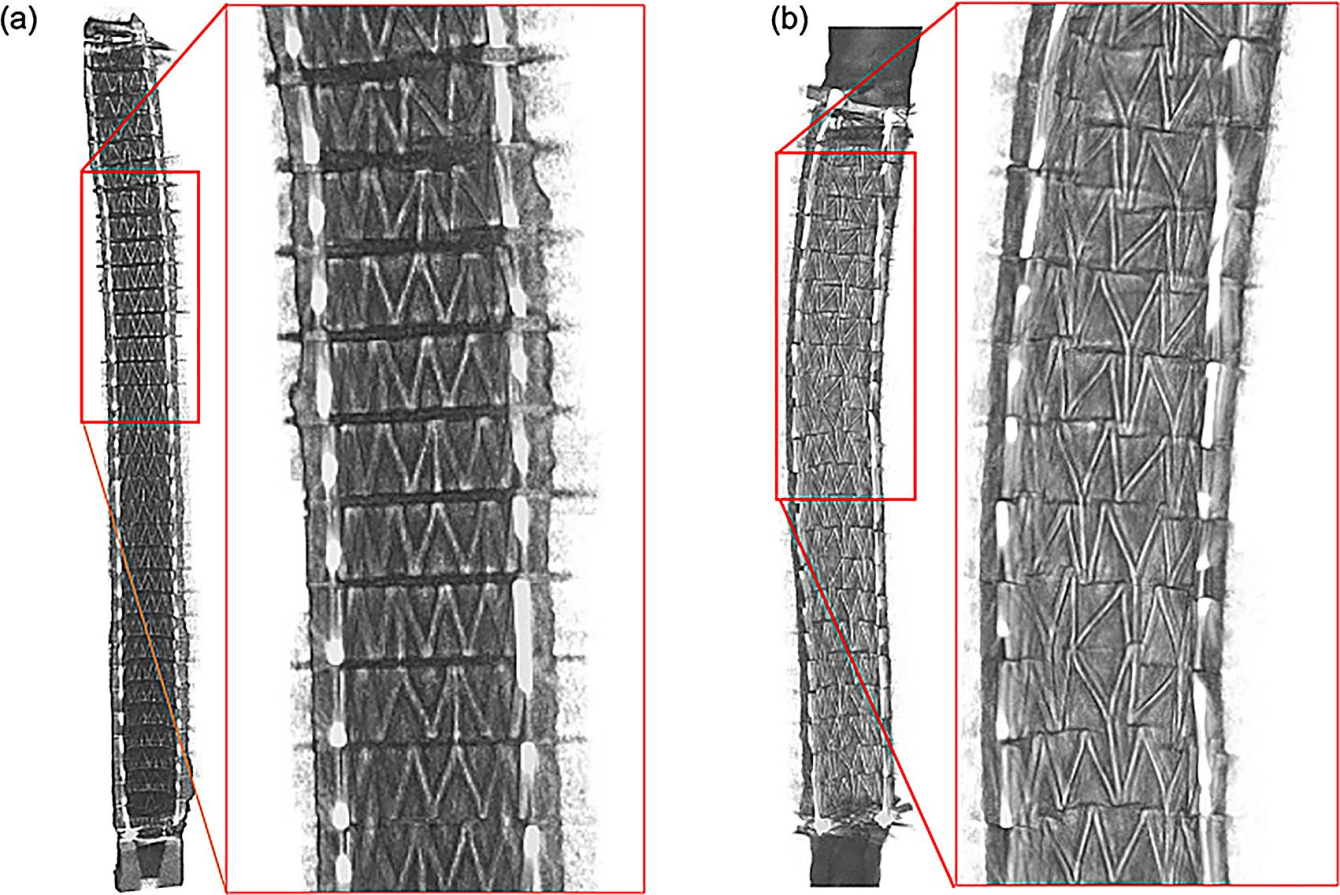
2D Micro CT imaging of the DES under (**a**) P2 and (**b**) P3 movements. A more enhanced version also shows the structure and placement of one section (red box) of the stents under these two parameters. P2 (Parameter 2): Pulsatile flow with 16.8° twist, 25° bend, 3.2 mm compression; P3 (Parameter 3): Pulsatile flow with 68° twist, 35° bend, 21 mm compression

**Fig. 6:**
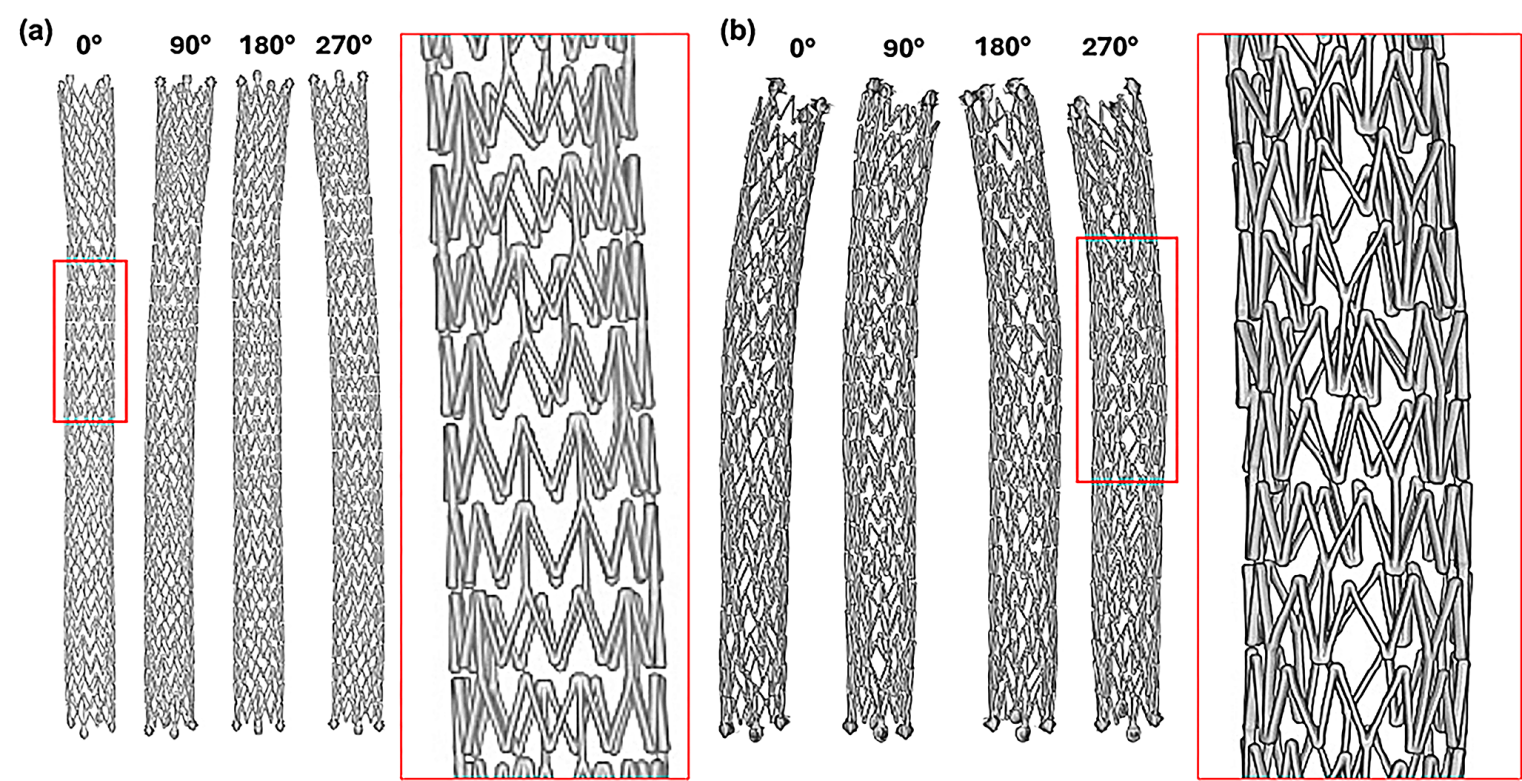
3D reconstruction of the DES from different angles under (**a**) P2 and (**b**) P3 moving conditions. A more enhanced version also shows the structure of the stent struts from one section (red box) in greater detail under these two conditions. P2 (Parameter 2): Pulsatile flow with 16.8° twist, 25° bend, 3.2 mm compression; P3 (Parameter 3): Pulsatile flow with 68° twist, 35° bend, 21 mm compression

**Fig. 7:**
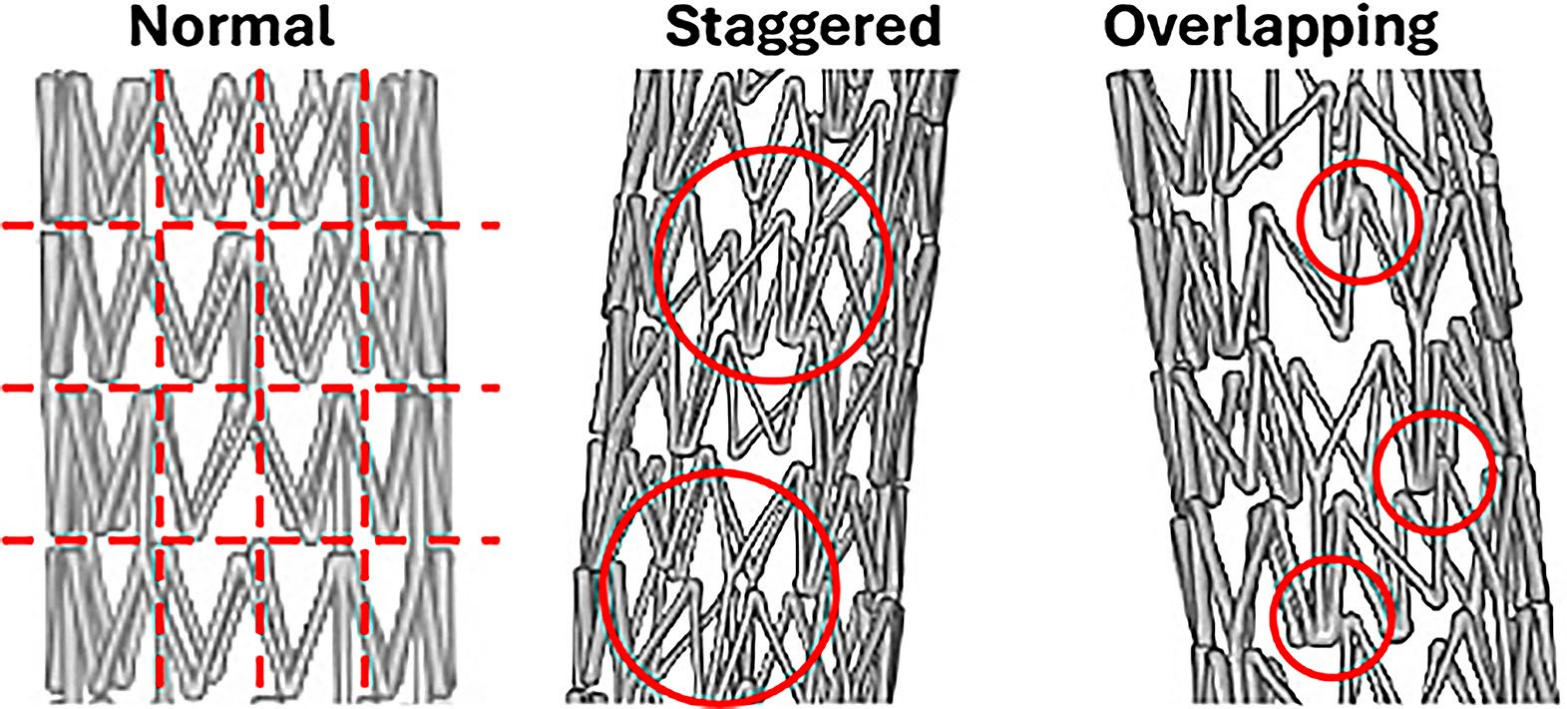
Representative images of various deformation of struts under various movements

Micro CT imaging was successful in showing the placement and structure of both stents inside the artery post-deployment (Fig. 5). The stents were fully expanded inside the arteries, which is to be expected for a self-expanding stent. However, adhesion to the arterial wall varied between the two arteries, which can be visually seen in comparing Fig. 5a with Fig. 5b. There were no protrusions or holes in the arteries, and there were no major artifacts with the stents being scanned inside of a 15 mL centrifugal tube filled with formalin. The 3D rendering also successfully showed the structure of both stents while still deployed in higher quality without the artery being visualized (Fig. 6). The imaging from the 4 different angles in Fig. 6a and b show deformation to one side toward the ends of the stents, although the deformation was more prominent in Fig. 6b (P3 movement). Due to the higher quality of the 3D rendering compared to the scan, the stent strut orientation was more visible than in Fig. 5, which revealed that the stent struts were noticeably more staggered in the P3 movement (Fig. 6b) than in the P2 movement group (Fig. 6a). Specifically, we evaluated each stent struts and categorized each strut as normal (stereotypical triangle shape), staggered (distorted strut), or overlapped (strut on top of neighboring stent; see Fig. 7). Results demonstrated that the stent exposed to P2 moving parameters had 18.9% staggered struts and 4.5% overlapped struts, whereas the stent exposed to P3 moving parameters had 63.6% staggered struts and 51.1% overlapped struts.

**Fig. 8:**
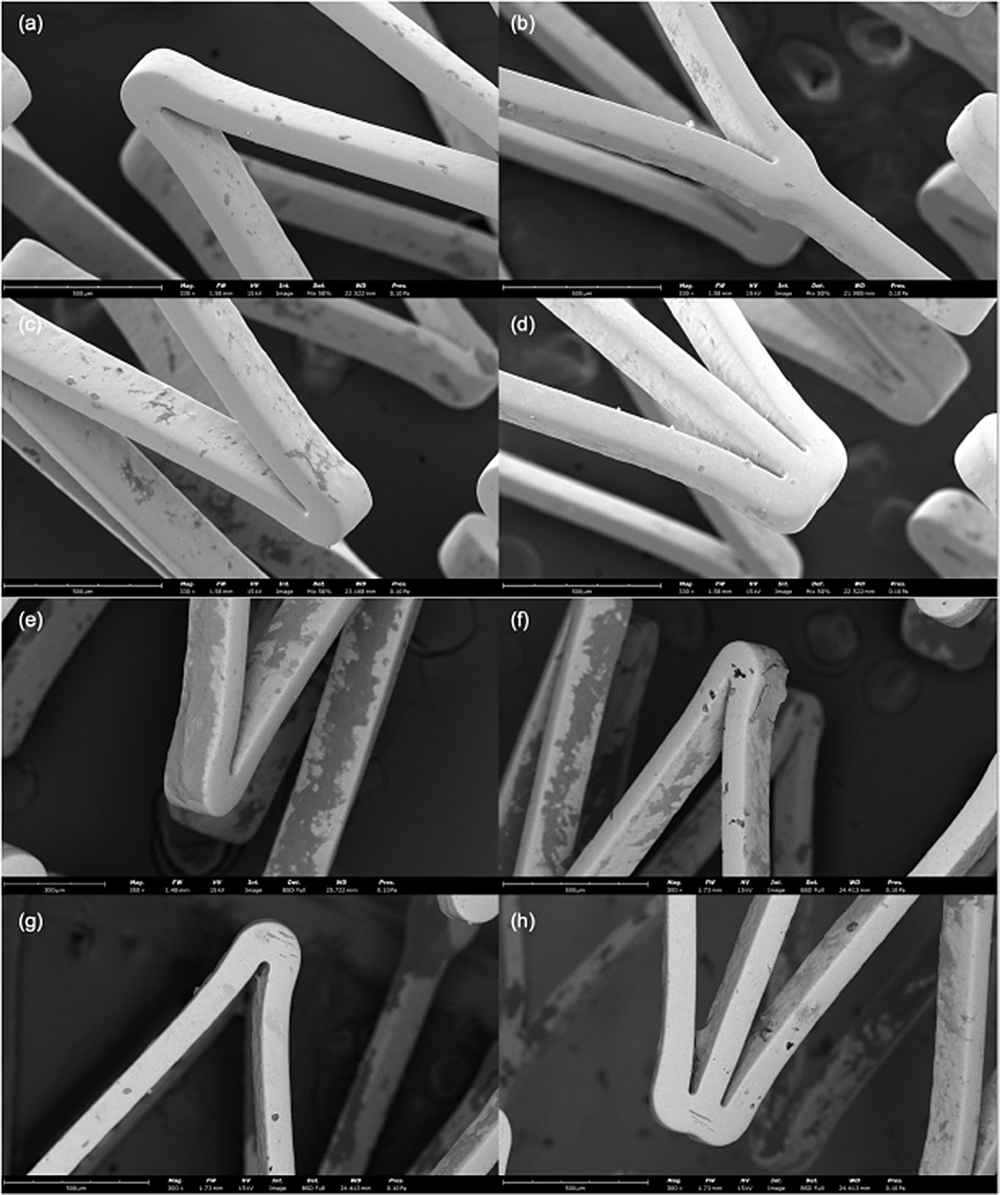
Scanning electron microscope images of deployed drug eluting stents. Representative SEM images of the Zilver drug eluting stent exposed to (**a** – **d**) P2 and (**e** – **h**) P3 moving parameter. P2 (Parameter 2): Pulsatile flow with 16.8° twist, 25° bend, 3.2 mm compression; P3 (Parameter 3): Pulsatile flow with 68° twist, 35° bend, 21 mm compression

While the Micro CT imaging was able to show the positioning of the stent inside the entire artery, SEM imaging was successful in showing the stent struts surfaces post-deployment on a micro-scale under both P2 and P3 moving parameters (Fig. 8). When the stents were removed from the arteries for imaging, they mostly reformed into their original shape due to their elastic nature. For that reason, the staggered orientation of the struts present in Fig. 6 were not present when using the SEM. The images showed slight coating disruptions to the nitinol in stents exposed to the P2 movement (Fig. 8a and c), but more extensive coating disruptions to the P3 movement (Fig. 8e and f). No images from either stent revealed fractures or breaks in the stent struts.

## Discussion

The goal of this study was to examine the effects of vascular motion on acute drug retention. Using a previously described ex-vivo peripheral simulating bioreactor system [19], we successfully quantified the effects of various degrees of vascular motion on drug absorption in two DCBs and one DES. Our results show that movement has a significant impact on acute drug absorption; specifically, PK analysis showed variation in the effects of vascular motion between the DCBs and DES as well as between the two DCBs, generally showing a decrease in drug absorption as the intensity of the movement increased. These results give insight into the treatment of PAD in areas of the body that are exposed to frequent and varied movement such as the FPA.

PAD is both widespread and costly, with over 237 million affected worldwide [21], and hospitalizations alone in the United States costing over $6 billion annually [23]. Both DCB and DES are the gold standard for the treatment of PAD and are often among the first forms of treatment utilized [24]. However, DCB and DES vary in their design and their mechanism of drug delivery. DCBs are meant to be deployed and expanded in the affected area to allow the drug to be absorbed into the arterial wall and then subsequently removed. DES are also deployed and expanded in the affected area; however, they are not removed after deployment and are instead kept in the affected area to continually release the drug in small quantities. Therefore, stents are subjected to the effects of movement more so than balloons, especially after deployment. Additionally, DCB and DES differ in their non-drug coatings: stents often use a polymer coating (though the Zilver stent is polymer-free [25]), whereas balloons use excipients, which enhance drug solubility, transfer, absorption, and retention [26]. These differences potentially contribute to the variation in drug absorption between DCB and DES that we demonstrated in this study (Fig. 4).

In addition to variation due to polymer or excipient coatings, DCB also vary in the type of excipient used. For example, the Lutonix DCB uses a mix of sorbitol and polysorbate excipients [27], whereas the IN.PACT DCB uses urea as an excipient [28]. These excipients can vary in their effectiveness in several ways, including the uniformity of drug dispersion on the balloon, the amount of drug loss, the efficiency and rate of drug transfer into the tissue, control of drug concentration, and rate of particulate degeneration [26, 29]. Additionally, the Lutonix DCB is coated using a pulverization method, whereas the IN.PACT DCB uses a micro-pipetting method [30]. Variation in coating methods can affect the amount of drug loss before deployment and the rate of drug absorption in the arterial wall [26]. Furthermore, the Lutonix and IN.PACT DCB differ in their dosage: the Lutonix DCB has a low-dose paclitaxel coating of 2 µg/mm^2^, and the IN.PACT DCB has a high-dose paclitaxel coating of 3.5 µg/mm^2^ [27, 28]. While some studies indicate no difference in outcome between high- and low-dose paclitaxel coatings [31], other studies show that high-dose paclitaxel coatings are more effective in the treatment of PAD [32]. Lastly, the formulation of paclitaxel on the Lutonix DCB is a hybrid of amorphous and crystalline, whereas the formulation of paclitaxel on the IN.PACT DCB is solely crystalline [30]. In comparison to the amorphous form, the crystalline form of paclitaxel has been shown to be more adhesive to the arterial wall and is retained within the wall at a higher concentration over a longer period of time [33, 34]. The differences in excipients, coating method, drug dosage, and drug crystallinity could play a role in explaining why vascular movement had a different effect on the retention of paclitaxel from the Lutonix and IN.PACT DCBs.

To evaluate the impact of vascular motion on drug retention, we selected one acute time point and two variations in vascular motion. First, the 24-hour acute time point was selected based on previous studies that show the concentration of paclitaxel on the Zilver stent decreases to around 5% of the original amount, and around 25% in the arterial tissue, after 24 h [35]. Next, the bending, twisting, and compression values in our three prescribed movement parameters (P1, P2, and P3) were based on ranges established from previous studies that examined the conformational change in the FPA in humans during different daily movement such as sitting, standing, and squatting [13]. Lastly, the number of cycles that were run during the 24-hour period were based on the average steps taken per day by U.S. adults [22]. The combination of a 24-hour acute time point, movement parameters based on real human movement, and cycles based on average activity validate our results from pharmacokinetic analysis.

As seen in the PK data (Fig. 4), there was no significant effect of movement on drug retention for the IN.PACT DCB, but both the P2 and P3 moving parameters significantly lowered drug retention in comparison to the P1 moving parameter (pulsatile flow only) for the Lutonix DCB. Similar to the Lutonix DCB, the Zilver DES also showed variation in the effect of vascular motion on drug retention between the different moving parameters; however, the pattern was different in the Zilver DES than in the Lutonix DCB. The mean paclitaxel concentration for the Zilver DES under P2 was 84.8 ± 32.7 ng/mg, a significantly greater retention compared to the mean concentration of 0.11 ± 0.06 ng/mg when exposed to the P3 moving parameter (*p* = 0.01; Fig. 4). These results suggest that the P3 movement parameter impacted the delivery or the retention of paclitaxel in the stented area after 24 h, in particular as the Zilver DES has no polymeric coating. Although the effects of polymers on the efficacy of DES are inconclusive [36, 37], polymers typically act to protect the drug from an initial burst release and allow for a more controlled release of the drug through physical and chemical mechanisms [26]. Therefore, if exposed to frequent movement, a polymer-free stent might allow the drug to be released and be absorbed into the arterial wall more rapidly, explaining the higher concentration of paclitaxel in the P2 when compared to the pulsatile only P1 movement at 24-hours. However, when a polymer-free stent is exposed to more intense movement, such as the P3 movement parameter, paclitaxel might fall off the stent and the arterial wall at a rate that is too fast to allow for adequate absorption, sending the drug downstream and away from the targeted site.

In parallel to the PK data, the Micro CT imaging showed that the more intense movement from the P3 movement parameter resulted in greater deformation of the stent inside the artery in comparison to the P2 movement (Fig. 5a and b). Specifically, more curvature of the stent and less direct contact of the stent strut to the inner arterial wall was visually observed. Additionally, the stent struts were less uniformly spaced and visually appeared staggered under the P3 movement versus the P2 movement (Fig. 5a and b). The 3D reconstruction of the stents further emphasizes the difference between these two movement conditions. An enhanced view of the stent structure shows that the stent exposed to the P3 movement was more deformed in comparison to the stent under the P2 movement (Fig. 6a and b). The staggered stent struts suggest that the twisting force was a significant contributor to stent deformation. Additionally, some of the struts in the stent exposed to the P3 movement protruded outside of the natural cylindrical shape of the stent, which was not present in the stent exposed to the P2 movement. Overall, the Micro CT imaging showed that the more intense bending, twisting, and compression motions of the P3 movement were contributing factors to arterial stent deformation post-deployment as compared to stents exposed to the P2 movement. These factors might contribute to the differences observed in paclitaxel retention in these stented tissues.

SEM imaging allowed for a higher quality visualization of the stent struts post-deployment (Fig. 8). There were no fractures in either stent; however, both stents amassed several micro-abrasions and other coating inconsistencies to the outer layer of the stent. As visually observed, the coating disruptions were more pronounced in the stent exposed to the P3 moving parameter as compared to the P2 movement (Fig. 8). These images suggest that the more intense movement of the P3 parameter may have contributed to the differences observed in paclitaxel absorption within the stented arterial segments.

Overall, these results highlight the impact of vascular motion on drug retention on routinely used clinical devices to treat peripheral arterial disease. Our data indicates that the acute loss of drug retention is not uniformly observed between various clinical devices and vascular motion can reduce drug retention in both a paclitaxel coated balloon, and a paclitaxel coated stent. Only the IN.PACT DCB did not show a significant reduction in drug loss following peripheral vascular motion. It is worth noting that that newer DCB designs are utilizing micro- and nano- drug particles that may provide better retention and new peripheral DES are utilizing polymer coatings. These new designs may overcome the limitations observed in this study, however testing similar to what has been shown here should be performed.

Our results support the concept that vascular movement alters drug retention of drug eluting intravascular devices; however, the study was limited to the use of culture medium in place of blood for the fluid in our bioreactor system. Pharmacokinetic studies were only limited to one time point, which does not provide any temporal data on drug retention. Additionally, our studies used healthy pig carotid arteries and not human diseased arteries, the intended target of these devices. We recognize that human diseased lesions are more complex and often include fibrosis, calcification, necrotic cores which may alter paclitaxel delivery and retention. Despite these limitations, our system has been validated in assessing drug delivery and retention using commercially available treatment methods in physiological conditions similar to those in vivo [38].

## Conclusions

This study is the first to examine the effects of vascular motion on acute drug retention using an ex vivo peripheral simulating bioreactor system in commercially available DCB and DES. Understanding how such movement impacts the absorption and retention of paclitaxel is essential to improving the treatment of PAD, especially in areas of the body subject to frequent and varied movement such as the FPA in which 30–40% of patients who undergo stent implantation experience restenosis within 2 years [39]. Future studies should isolate the specific factors of vascular movement that impact the utilization of DCB and DES for the treatment of PAD. Understanding these mechanisms will better elucidate how to improve long-term patency in this critical area of interventional cardiology.
